# COVID-19: dealing with a potential risk factor for chronic neurological disorders

**DOI:** 10.1007/s00415-020-10131-y

**Published:** 2020-08-27

**Authors:** Tommaso Schirinzi, Doriana Landi, Claudio Liguori

**Affiliations:** 1 Neurology Unit, University Hospital of Rome Tor Vergata, Viale Oxford 81, 00133 Rome, Italy; 2 Multiple Sclerosis Clinical and Research Unit, University Hospital of Rome Tor Vergata, Viale Oxford 81, 00133 Rome, Italy; 3 Sleep Medicine Centre, Department of Systems Medicine, University of Rome Tor Vergata, Viale Oxford, 81, 00133 Rome, Italy; 4 Neurology Unit, University Hospital of Rome Tor Vergata, Viale Oxford, 81, 00133 Rome, Italy

**Keywords:** Parkinson’s disease, Multiple sclerosis, Narcolepsy, SARS-CoV2, COVID-19, Neuroinflammation, Risk factors, Neurodegeneration, Demyelination, Sleepiness

## Abstract

SARS-CoV2 infection is responsible for a complex clinical syndrome, named Coronavirus Disease 2019 (COVID-19), whose main consequences are severe pneumonia and acute respiratory distress syndrome. Occurrence of acute and subacute neurological manifestations (encephalitis, stroke, headache, seizures, Guillain–Barrè syndrome) is increasingly reported in patients with COVID-19. Moreover, SARS-CoV2 immunopathology and tissue colonization in the gut and the central nervous system, and the systemic inflammatory response during COVID-19 may potentially trigger chronic autoimmune and neurodegenerative disorders. Specifically, Parkinson’s disease, multiple sclerosis and narcolepsy present several pathogenic mechanisms that can be hypothetically initiated by SARS-CoV2 infection in susceptible individuals. In this short narrative review, we summarize the clinical evidence supporting the rationale for investigating SARS-CoV2 infection as risk factor for these neurological disorders, and suggest the opportunity to perform in the future SARS-CoV2 serology when diagnosing these disorders.

## Introduction

In December 2019, a novel coronavirus, named severe acute respiratory syndrome-coronavirus 2 (SARS-CoV2), emerged from China and spread worldwide as pandemic. SARS-CoV2 infection is responsible for a heterogeneous clinical syndrome, leading to severe pneumonia and acute respiratory distress syndrome (ARDS), titled coronavirus disease 2019 (COVID-19). The occurrence of neurological manifestations, including encephalitis, stroke, headache, seizures, Guillain–Barrè syndrome, is increasingly reported in patients with COVID-19 [1–4]. Although these neurological manifestations of COVID-19 suggest a possibly acute or subacute neuropathogenicity of the virus, the risk of long-term neurological sequelae in patients affected by SARS-CoV2 is not understood and currently debated [5, 6].

Available data on COVID-19 currently disclosed that SARS-CoV2 can induce, directly or indirectly, a number of clinical manifestations and immune–inflammatory events, including viral–host interactions, that might shape pathogenic mechanisms underlying common chronic neuroinflammatory and neurodegenerative disorders [2, 5, 6].

In this review, we will specifically discuss the biological events possibly initiated by SARS-CoV2 infection potentially overlapping with etiological mechanisms featuring Parkinson’s disease (PD), multiple sclerosis (MS), or narcolepsy. Building on these evidences, we will highlight the need to monitor patients affected by COVID-19 who can develop PD, MS, or narcolepsy as long-term neurological consequences of the infection.

## Parkinson’s disease

PD is the second most common neurodegenerative disorder, characterized by progressive motor and non-motor disturbances, due to the loss of dopaminergic cells in the substantia nigra pars compacta (SNpc) and the accumulation of α-synuclein (α-syn)-positive Lewy bodies [7–9]. The relationship between viral infections and PD has its roots in the early twentieth century, when a number of post-encephalitic parkinsonism were observed following an influenza outbreak. Aside from this historical event, infectious diseases, including viral infections, have been demonstrated increasing the risk for PD by 20% [10]. The mechanisms underlying this association may imply a direct neuronal injury due to the central nervous system (CNS) invasion by viruses and subsequent loss of dopaminergic cells into the SNpc. Indeed, it has been recently demonstrated in *Rag* knockout mice that H1N1 Influenza-A virus infection inhibits protein degradation at autophagosome–lysosome system level and precipitates α-syn accumulation [11]. Further experimental evidence showed that Influenza-A virus disrupts mitochondrial activity and increase oxidative stress [12, 13], whereas hepatitis C virus impairs dopaminergic transmission and affects the blood–brain barrier (BBB) integrity [10]. Therefore, viral infections may intervene in cellular pathways critical for PD pathogenesis, probably contributing to the initiation of the disease [8, 9, 14–17].

Although the CNS colonization by SARS-CoV2 has been proven, the consequences on neurons at a molecular level have been only hypothesized [5, 6]. However, it is interesting to note that the virus may affect brain areas particularly involved in early phases of PD neurodegeneration. Many patients with COVID-19 indeed complained of anosmia and ageusia [18], which are two classical prodromal features of PD [19]. Actually, SARS-CoV2 might invade the brain through the olfactory tracts and spread towards the piriform and infralimbic cortex, the basal ganglia and the brainstem [18]. Neuropathological evidence suggests that, in PD, Lewy body accumulation is primarily localized in the olfactory pathway, and then propagates to other brain structures following olfactory system connections causing neuronal degeneration [19, 20]. This potential overlap between the SARS-CoV2 propagation and the spreading of PD neuropathology is particularly alarming if we consider that some patients with COVID-19 do not recover (or partially recover) smell sense [18], thus, indicating a possible neuronal injury that in turn might trigger the synucleinopathy cascade [21]. Aside from the direct invasion of CNS, SARS-CoV2 might increase the risk for PD because of the induction of a systemic inflammatory state [22]. Cytokine production is fundamental in the immunological response to viruses. However, an excessive and dysregulated release of interferons (IFNs), interleukin (IL)-1β, IL-6, tumour necrosis factor (TNF) and chemokines (C–C motif chemokine ligand, CCL-2, CCL-3, and CCL-5), shaping the so-called cytokine storm, can be deleterious, causing an immune-mediated attack to human organs [23]. COVID-19 patients present a systemic inflammatory state, as demonstrated by the significant increase of C-reactive protein (CRP), IL-6, IL-8, IL-10, IL-2R, and ferritin blood levels [24]. Similar profile of peripheral inflammation is notably observed in PD patients, who exhibit higher blood levels of CRP and proinflammatory cytokines (IL-6, TNF, IL-1β and IL-2) [25, 26], directly correlated with clinical severity [27]. The inflammatory activation due to COVID-19 may thus disrupt the systemic homeostasis at the CNS level, where it could trigger and feed initial steps of synucleinopathy, favouring PD onset, as compelling experimental evidence suggests [22, 28].

COVID-19 is also responsible for gastrointestinal symptoms [29], and SARS-CoV2 RNA has been tracked in the faeces of infected patients indicating an intestinal localization of the virus. A recent study demonstrated that enterocytes represent major target cells of SARS-CoV2 reacting to the infection with a strong inflammatory response [30]. These findings might further highlight the role of COVID-19 as a potential risk factor for PD. In fact, an experimental intestinal infection was able to turn *PINK1* asymptomatic mouse model into a fully penetrant model, with levodopa-responsive motor symptoms, probably trough an immune-mediated multisystem mechanism [31]. Moreover, SARS-CoV2 intestinal infection may alter gut microbiota and gut physiology overall [32], influencing all factors providing the “peripheral” contribute to PD pathogenesis and progression [33].

Finally, it should be also considered psychiatric comorbidity of COVID-19. Actually, patients can develop depression, anxiety and fatigue, which may have both psychological and organic causes [34]. Regardless of the cause, mood disorders are associated with neuroinflammation and often exert detrimental effects on CNS, contributing to neurodegeneration [35]. Hence, COVID-19 definitely represents a stressful event that may have a role in triggering PD [36].

## Multiple sclerosis

MS is a chronic immune-mediated disease of the CNS whose pathological hallmark is focal demyelination associated with various degrees of neurodegeneration [37]. Complex immunological dysfunction—involving peripheral T and B lymphocytes and resident CNS immune cells—represents the immunological substrate for MS development and progression [38]. The intermittent aberrant activation of self-reacting immune cell subsets results in their transmigration across the BBB into the CNS, where they induce demyelinating and, ultimately, neuronal damage manifesting as clinical relapse and disability accumulation. The aetiology of the disease, as well as its periodic relapses, is not established yet, but environmental triggers acting on susceptible individuals are implicated. For over a century, since Pierre Marie initial intuition in 1884, MS was believed to be caused by infectious agents and many viruses, including coronavirus, have been proposed as potential candidates [39]. Viral infection contributes to demyelination through several mechanisms such as molecular mimicry, bystander inflammatory damage or direct oligodendrocyte infection [39]. MS onset may occur long after acute infection as consistently demonstrated for by Epstein–Barr virus (EBV) [40]. Infectious mononucleosis by EBV supervening during the early adulthood, in fact, is an established risk factor for further MS development [41–45]; moreover, compelling evidence shows that almost all subjects with MS have positive serology for EBV. The “prime/challenging” theory has been proposed to explain the delay between early infection and MS onset; according to this assumption, the initial infection, such as by EBV, would prime autoreactive cells in susceptible individuals via molecular mimicry and bystander activation, setting up a fertile-field. Further infection by other microorganism, or even reactivation of EBV under favouring circumstances, will activate the preexisting autoreactive cells leading to inflammatory demyelination [40, 46]. Studies in MS patients infected by SARS-CoV2 are ongoing aiming at identifying the effects of iatrogenic immune modulation/suppression on the severity of infection [47, 48]. Nevertheless, the effect of the virus on MS-related inflammatory activity has not been investigated yet, but few cases of acute inflammatory demyelinating disorder have been already described. It would not be surprising that SARS-CoV2 might act as “priming” or “challenging” infectious agent in “primed” individuals. Moreover, in individuals with MS, autoreactive T cells able to recognize both viral and myelin antigens have been found [49]. Additionally, SARS-CoV2 infection is associated with peripheral lymphopenia in more than 80% of patients with COVID-19. Lymphopenia is sustained by a predominant decrease of CD3 + , CD8 + , and CD4 + T cell counts, while B cells and NK are only mildly affected [50]. Patients infected by SARS-CoV during the 2002–04 outbreak recovered normal T lymphocytes count in about 2 months in the majority of case, and more rarely the recovery took more than 12 months [51]. Sequestration in the lung, intestine and other tissues, and senescence and exhaustion of the anti-viral CD8 response [50, 52], explain this selective immunodepletion. We can speculate that defective anti-viral CD8 immunological response may reduce immunosurveillance on other latent pathogens potentially able to trigger MS or other post-infectious demyelinating disorders, such as Guillain–Barrè syndrome or its variants [53]. Co-infection with EBV, in fact, has been observed in patients affected by COVID-19, mainly in those with lower CD4/CD8 ratio [54]. Nevertheless, unbalance of peripheral lymphocyte subsets induced by COVID-19, and in particular B cell overshooting, may hypothetically represent an additional risk for MS relapses in patients with pre-existing diagnosis, as observed in similar immunological framework [55]. The “cytokine storm” in response to the SARS-CoV2 infection may promote a switch toward a pro-inflammatory status of T cell subsets, such as Th17, which are implicated in MS pathogenesis [56]

COVID-19 may indeed trigger MS or its clinical manifestation also through other mechanisms. In MS, intestinal dysbiosis and changes in intestinal permeability are increasingly recognized as modulators of neuroinflammatory mechanisms through the so-called gut–brain axis [57]. Therefore, the alteration of the intestinal barrier and microbiota induced by SARS-CoV2 may enhance autoreactive response (as previously mentioned).

Finally, it is worth noting that SARS-CoV2 is able to directly infect the CNS via olfactory pathway or hematogenous route using the angiotensin-converting enzyme receptor type 2 (ACE2) expressed in the CNS and in the vascular endothelium [6].

Coronaviruses, such as mouse hepatitis virus, may invade neurons and oligodendrocytes, establish a persistent infection of astrocytes and locally activate and immortalize microglial cells causing brain and spinal demyelination featuring MS, as observed in animal models and humans [58–61]. Moreover, strains of human coronavirus have been found in brain autoptic specimens of patients with MS [62]; additionally, MS patients show higher intrathecal antibody synthesis against coronaviruses than matched controls [45].

Building on these evidences, MS may result from previous SARS-CoV2 infection due several mechanisms: (1) a “challenging” effect of the virus in susceptible subjects previously exposed to priming pathogens; (2) unbalance of peripheral lymphocyte subsets and massive cytokine release producing a pro-inflammatory environment and triggering autoimmune reactions; (3) induction of post-infectious demyelinating events associated with direct CNS invasion and microglial reaction.

## Narcolepsy

Narcolepsy is a rare sleep disorder featured by excessive daytime sleepiness (EDS) and REM sleep-associated symptoms, such as cataplexy (loss of muscle tone triggered by strong emotions), hypnagogic/hypnopompic hallucinations, and sleep paralysis. The prevalence of narcolepsy in 2016 was of 44.3 per 100,000 persons [63]. The main increase in narcolepsy diagnosis was in particular evident following Influenza-A H1N1 pandemic in 2009, and was evident in both patients affected by Influenza-A H1N1 and in patients vaccinated against this virus with Pandemrix (an adjuvanted vaccine) [64]. International classification of Sleep Disorders—3rd Edition classifies narcolepsy into two types, namely narcolepsy types 1 and 2 (NT1/2) [65]. The main clinical difference between these two forms of narcolepsy is the occurrence of cataplexy, which is the result of orexin (OX) neuron degeneration [65]. The OX system degeneration results in the not detectable levels of OX in the cerebrospinal fluid (CSF), consisting of the main diagnostic feature of NT1 [66]. Conversely, in NT2, the partial degeneration of OX neurons corresponds to normal CSF OX levels [67].

The main pathogenic causes of narcolepsy have been exclusively supposed with different levels of evidence since OX neuron degeneration remains a not-well-explained phenomenon. Several lines of evidence suggest that narcolepsy arises from the interaction of genetic, environmental and triggering factors, which leads to an immune-mediated selective loss or dysfunction of OX neurons in the brain lateral hypothalamus. Briefly, as summarized by Bassetti and coauthors [68], genetic factors (especially HLA-DQB1*06:02 positivity) are a strong predisposition to narcolepsy. Ensuing this genetic susceptibility, environmental exposures to bacterial and viral infections may alter or trigger the immune system reaction that in turn may attack the OX neurons. Several researches have been performed to understand the cascade of events leading to OX system and involving the different subsets of immune cells (B cells, T CD4 + and T CD8 + cells) [69, 70]. Not significant results have been achieved regarding the detection of specific autoantibodies produced by B cells [71]; conversely, T cells seem to have direct and indirect effects on OX neurons. In particular, in 2018, autoreactive CD4 + and CD8 + T cells targeting antigens expressed by OX neurons have been documented in patients with NT1 or NT2 [72]. This research highlighted the role of T cells in the pathogenesis of narcolepsy; however, the lack of proliferation of T cell clones in response to H1N1 influenza vaccine does not permit to achieve a definite conclusion [72]. Therefore, although widely supposed the role of T cells in the pathogenesis of narcolepsy, the chain of events producing OX neuron degeneration has not been completely identified. Finally, the increased levels of specific cytokines (TNF and IL-6 among others) further support the evidence of an inflammatory and immune response in patients with narcolepsy since the very early phases of the disease [73]. The reduction of CSF ß-amyloid_42_ levels in patients with narcolepsy near to disease onset has been also associated with the brain inflammatory response [74–76]. Moreover, other proofs of the activation of the immune system have been documented in patients with NT1, also with long-lasting disease [77].

The SARS-CoV2 viral outbreak may also present a unique opportunity to better understand the association between immune system activation and the development of autoimmune conditions such as narcolepsy [78]. Considering the non-haematological routes of infection, SARS-CoV2 can migrate from the olfactory bulb to hypothalamus and affect the OX neurons [78]. In keeping with this hypothetical model of CNS damage, the olfactory bulb may represent a link between environmental agents (such as SARS-CoV2) and narcolepsy, in patients with a genetic predisposition [79]. The olfactory bulb, in fact, provides an efficient port for neuroinvasion [80]. Neurotropic, but also non-neurotropic, viruses may use this gateway to enter the CNS using the BBB disruption caused by the activated inflammatory processes [80]. Moreover, the inflammatory response (in particular proinflammatory cytokines) can enhance BBB permeability promoting the transendothelial migration of T cells (activated against the virus), which can damage the OX hypothalamic neurons [81]. The documentation of olfactory dysfunction in patients with narcolepsy can reinforce this hypothesis and highlight the role of olfactory bulb in the pathogenic mechanisms of narcolepsy [82].

Taking these hypotheses into account, the main message of this review to sleep medicine clinicians and researches is to consider SARS-CoV2 infection as a possibly triggering event leading to narcolepsy. The previous experience of Influenza-A H1N1 infection and vaccination should raise the opportunity to monitor subjects affected with COVID-19 also after resolution of the infection since the occurrence of EDS (in same cases already present during the infection) may represent a preliminary manifestation of OX dysfunction.

## Conclusion

Both retrospective analysis achieved by reviewing clinical charts of patients with COVID-19 and prospective observational studies [2, 83, 84] provided compelling evidence on the CNS involvement during SARS-CoV2 infection, which definitely supports the hypothesis of a neuropathogenic effect of the virus. Early experimental data on SARS-CoV2 and existing literature about other coronaviruses allow supposing several mechanisms of neuroinvasion of the virus, including the trans-synaptic spread from peripheral nerves, the BBB passage mediated by ACE2 receptors or abnormal permeability, and the “Trojan horse” strategy due to the brain entrance of immune cells infected through ACE2 receptors [2, 6].

**Fig. 1:**
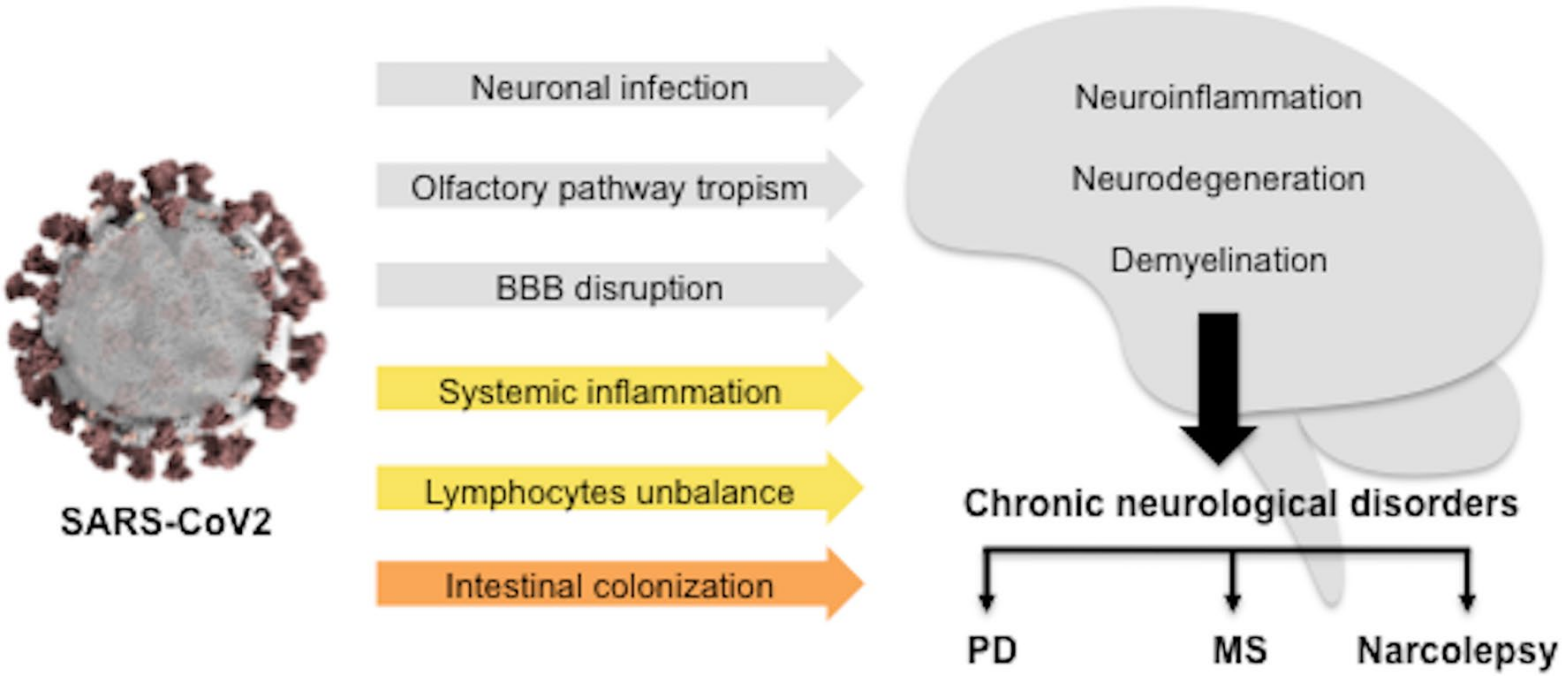
SARS-CoV2-induced mechanisms for neuropathogenicity. The scheme represents direct and indirect effects of COVID-19 that overlap with defined pathogenic mechanisms of common and rare chronic neurological disorders, suggesting its potential role as risk factor

This brief narrative review summarized the mechanisms of CNS affection during SARS-CoV2 infection, which include different pathways and pathogenic cascades, concluding in chronic neuroinflammatory or neurodegenerative processes that typically underlie both common (PD and MS) or rare (narcolepsy) neurological diseases. In addition to direct neuronal injury, we also highlighted how SARS-CoV2 might have a role in the successive development of these chronic neurological disorders because of the activation of systemic inflammatory response, favouring a culprit unbalance in the immune system or affecting other critical players of neurodegeneration and neuroinflammation, such as BBB integrity and gut–brain axis (Fig. 1).

Although long-term neuropathogenic effect of SARS-CoV2 has not yet been proven in experimental settings, available knowledge on both COVID-19 clinical events and established pathophysiological dynamics of chronic neurological disorders lead us to look at SARS-CoV2 infection as a potential trigger or risk factor for neurological disorders.

In conclusion, prospective neurological follow-up of both COVID-19 survivors and asymptomatic infected individuals, and case–control observational studies are mandatory to establish the effective long-term neuropathogenicity of the virus and achieve early diagnosis and timely therapeutic interventions. On the other hand, COVID-19 should be considered a critical anamnestic cue and serology for SARS-CoV2 infection can be planned when approaching patients with neuroinflammatory, neurodegenerative, or sleep disorders.
